# Downregulation of iNOS, IL-1*β*, and P2X7 Expression in Mast Cells via Activation of PAR4 Contributes to the Inhibition of Visceral Hyperalgesia in Rats

**DOI:** 10.1155/2018/3256908

**Published:** 2018-05-09

**Authors:** Yanli Hao, Hao Niu, Shuhong An, Ming Wang, Zhaojin Wang

**Affiliations:** ^1^Institute of Basic Medical Science, Taishan Medical University, Taian, China; ^2^Department of Human Anatomy, Taishan Medical University, Taian, China

## Abstract

Protease-activated receptor 4 (PAR4) is implicated in the inhibition of visceral hyperalgesia. In the present study, the effects of PAR4 activation on visceral hypersensitivity and expression of inflammatory mediators, including interleukin-1*β* (IL-1*β*), P2RX7 purinergic receptor (P2X7), inducible nitric oxide synthase (iNOS), and tryptase, in mast cells (MCs) were investigated via in vivo and in vitro studies. The numbers of tryptase-positive MCs with extensive PAR4, P2X7, and iNOS expression were increased in the colons of visceral hyperalgesia rats compared with controls. Intracolonic administration of PAR4-activating peptide (PAR4-AP) significantly attenuated the visceral hypersensitivity to colorectal distention and reduced the iNOS, IL-1*β*, P2X7, and tryptase protein and mRNA levels in the colonic mucosa. Treatment of rat bone marrow MCs (BMMCs) with PAR4-AP also reduced the iNOS, IL-1*β*, P2X7, and tryptase protein and mRNA levels. ERK1/2 and p38 activators (t-butylhydroquinone, tBHQ, and U-46619) reversed the suppressive effect of PAR4 activation on IL-1*β* and iNOS expression, whereas ERK1/2 and p38 inhibitors (PD98059 and SB203580) reversed the suppressive effect of PAR4 activation on P2X7 and tryptase expression. Our results indicate that the downregulation of inflammatory mediators, including iNOS, IL-1*β*, P2X7, and tryptase, in MCs that are mediated by PAR4 activation could inhibit visceral hyperalgesia via the mitogen-activated protein kinase (MAPK) signal pathway.

## 1. Introduction

Accumulating evidence suggests that mast cells (MCs), especially mucosal MCs, have crucial roles in the regulation of intestinal motility, visceral sensitivity, mucosal inflammation, the permeability of the epithelial barrier, and the immune system in irritable bowel syndrome (IBS) [1, 2]. Increased numbers of activated MCs and increases in MC products have been observed throughout the gastrointestinal mucosa of IBS patients [3, 4]. The soluble mediators released by activated MCs, particularly proinflammatory mediators and cytokines such as tryptase and interleukin-1*β* (IL-1*β*), contribute to visceral hyperalgesia [5, 6].

Protease-activated receptor 4 (PAR4) is a member of the G-protein coupled receptor family and may mediate an antinociceptive effect, which would indicate new roles in the modulation of visceral hyperalgesia and hypersensitivity [7, 8]. Previous research has demonstrated that the activation of PAR4 inhibits colonic hypersensitivity through the suppression of the excitability of colonic sensory neurons and their primary afferent responses to pronociceptive mediators [8, 9]. PAR4 is highly expressed in MCs in the colons of IBS patients [10, 11]. MCs are efficient producers of many key inflammatory cytokines in response to a variety of stimuli, including as nitric oxide (NO)/inducible NO synthase (iNOS), ATP-reactive P2RX7 purinergic receptor (P2X7), and inflammatory cytokines [12, 13]. Several studies have reported that mitogen-activated protein kinases (MAPK), such as extracellular signal-regulated protein kinase 1/2 (ERK1/2) and p38 MAPK (p38), are crucial mediators of inflammation in inflammatory bowel disease (IBD) [14, 15]. Recently, we reported that PAR4 activation suppresses the inflammatory cytokines associated with the phosphorylation of ERK1/2 and p38 in MCs [16]. However, the function of PAR4 on MCs in visceral hypersensitivity is relatively unknown. Therefore, a better understanding of the role of PAR4 activation on MCs in the gut in visceral hyperalgesia is needed.

In the present study, we examined potential influence of PAR4 activation on colonic sensations in a visceral hyperalgesia rat model and the expressions of iNOS, P2X7, IL-1*β*, and tryptase in MCs, which might regulate sensitization and the consequent heightened pain behavior in IBS. We also investigated whether the activation of PAR4 affects the MAPK pathway, which involved in the expressions of iNOS, P2X7, IL-1*β*, and tryptase in MCs.

## 2. Materials and Methods

### 2.1. Induction of Chronic Visceral Hyperalgesia

The rat model of visceral hyperalgesia was induced as previously described [17]. Briefly, daily 60 mmHg colorectal distension (CRD) was performed on neonatal rats between 8 and 21 postnatal days after birth. The distention was applied using a vascular reconstruction balloon (length 20.0 mm, diameter 2.5 mm) that was inserted into the descending colon through the rectums of awake rats. The balloon was quickly distended at 60 mmHg for 1 min and then deflated and withdrawn. The control rats received the same procedure except for the CRD. The experiments were performed in these rats at the age of at least 8 weeks old.

### 2.2. Intracolonic Administration

The visceral hyperalgesia rats received an intracolonic administration of 100 *μ*g PAR4-activating peptide (PAR4-AP) or control peptide diluted in 150 *μ*l 0.9% NaCl. The visceral sensitivity measurements began 60 min following the end of the intracolonic administration.

### 2.3. Colorectal Distension and Electromyographic Recording

We used the electromyographic (EMG) recordings of the external oblique muscle and abdominal withdraw reflex (AWR) scores to evaluate visceral hypersensitivity 8 weeks after treatment according to the responses of rats to CRD as described previously [17, 18]. Briefly, the rats were anesthetized with ether, and CRD was performed via the insertion of a plastic balloon (5 cm) into the descending colon and rectum to 1 cm from the anus. Silver bipolar electrodes were inserted above the inguinal ligament on the side of the external oblique muscle 1.5 cm away from the midline. After the rats recovered from the inhalation anesthesia, the balloon was inflated to 20, 40, 60, and 80 mmHg for 10 s followed by 4 min of rest. The magnitude of the EMG activity was measured with a RM6240BD multichannel physiological signal acquisition and processing system (Chengdu, China). The EMG signals were amplified, filtered (×10,000, 300–5000 Hz), digitized, and rectified as previously detailed [18]. The area under the curve (AUC) values of the EMGs during the first and second distensions was computed, and the basal AUC was subtracted to obtain the net AUC in response to CRD.

### 2.4. Bone Marrow MC (BMMC) Preparation and Induction

BMMCs were cultured from the bone marrow cells (BMCs) of rats as previously described [19]. Briefly, BMCs were cultured for up to 10 weeks in enriched RPMI-1640 medium (containing 100 U/ml penicillin, 100 *μ*g/ml streptomycin, 25 mmol/l HEPES, 2 mmol/l L-glutamine, 1 mmol/l sodium pyruvate, 0.1 mmol/l nonessential amino acids, 0.05 mmol/l *β*-ME, and 10% FBS) in the presence of both recombinant rat IL-3 (5 ng/ml, R&D Systems Inc.) and recombinant mouse stem cell factor (SCF, 5 ng/ml, PeproTech). The nonadherent cells were hemidepleted twice each week with enriched medium containing the cytokines mentioned above. After 3 weeks, >98% of the cells in the culture were MCs as determined by staining with toluidine blue.

### 2.5. Drug Administration

Cultured BMMCs that were harvested at 4 weeks were continuously stimulated with PAR4-AP (100 *μ*mol/l) for 60 min. To assess the possible effects of ERK1/2 and p38 on the regulation of tryptase, iNOS, P2X7, and IL-1*β* expression following PAR4-AP stimulation, PD98059 (10 *μ*mol/l), SB203580 (10 *μ*M), tBHQ (50 nmol/l), or U-46619 (10 nmol/l) was added to six-well plates 60 min prior to the addition of PAR4-AP. Cells stimulated with PAR4 control peptide (100 *μ*mol/l) without ERK1/2 or p38 inhibitors or activators were used as controls. Sister six-well plates of BMMC cultures were used to compare the control cells with the cells that were treated with PAR4-AP.

### 2.6. Immunohistochemistry

All colonic samples from the visceral hyperalgesia rats and controls were fixed in buffered 4% paraformaldehyde for 30 min. After overnight cryoprotection in 20% buffered sucrose, 8-*μ*m-thick cryostat sections were mounted on poly-L-lysine-coated slides. The sections were preincubated with 1% BSA, 0.5% Triton X-100, and 10% normal donkey serum for 60 min at room temperature. The slides were incubated in a moist chamber with AA1 mouse monoclonal antibody (anti-mast cell tryptase antibody; 1 : 500, Abcam) at 4°C overnight. The slides were then washed and incubated with horseradish peroxidase- (HRP-) labeled goat anti-mouse antibody (1 : 100) for 45 min at 37°C. Next, the slides were developed in 0.05% freshly prepared 3,3′-diaminobenzedine (DAB) solution with 0.03% hydrogen peroxide for 8 min and then counterstained with toluidine blue, dehydrated, air-dried, and mounted in neutral resins. Immunoreactivity was quantified as previously detailed [13].

### 2.7. Western Blotting

Tissues or cultured BMMCs were lysed, and the protein was extracted. The protein lysate from each sample was separated electrophoretically on a sodium dodecyl sulfate-polyacrylamide gel and then transferred to a polyvinylidene fluoride (PVDF) membrane. After blocking with 5% nonfat milk in TBS-T (containing 0.1% Tween-20) for 2 hrs, the membranes were incubated with iNOS, P2X7, IL-1*β* (Novus Biologicals), and tryptase (AA1) antibodies in 5% nonfat milk in TBS-T overnight at 4°C. After washes with TBS-T, the membranes were incubated with the appropriate secondary antibodies for 2 hrs. The results were visualized using an ECL chemiluminescence system. GAPDH rabbit mAb antibody (Cell Signaling Technology) was also used as a probed control to ensure the loading of equivalent amounts of the sample proteins. The band densities were compared in TotalLab software (version 2.01; Bio-Rad, Hercules, CA).

### 2.8. Quantitative Real-Time Polymerase Chain Reaction (qRT-PCR)

Total RNA was isolated from the colonic tissues or BMMCs using TRIzol reagent (Invitrogen). The RNA concentrations were determined spectrophotometrically. Subsequently, cDNA was synthesized using a cDNA synthesis kit (Invitrogen) according to the manufacturer's instructions. The synthetic oligonucleotide primer sequences were as follows: P2X7: 5′-TTACGGCACCATCAAGTGGA-3′ (sense) and 5′-GCAAAGGGAGGGTGTAGTCG-3′ (antisense); iNOS: 5′-TTCAGTATCACAACCTCAGCAAG-3′ (sense) and 5′-TGGACCTGCAAGTTAAAATCCC-3′ (antisense); IL-1*β*: 5′-ATGATGGCTTATTACAGTGGCAA-3′ (sense) and 5′-GTCGGAGATTCGTAGCTGGA-3′(antisense); tryptase: 5′-TACCGCTATGTCCCCAAGGA-3′ (sense) and 5′-GAGGGACACAAGTGGTCAGG-3′ (antisense); and *β*-actin: 5′-ATCGTGCGTGACATTAAGGAGAAG-3′ (sense) and 5′-AGGAAGGAAGGCTGGAAGAGTG-3′ (antisense). Following reverse transcription, quantitative RT-PCR was performed using a 7300 real-time PCR system (Applied Biosystems, Foster City, CA, USA) according to manufacturer's instructions. In the control reactions, the reverse transcriptase was omitted. A comparative cycle threshold fluorescence (ΔCt) method was used, and the relative transcript amount of the target gene was normalized to that of *β*-actin using the 2^−ΔΔCT^ method. The final results of the real-time PCR are expressed as the ratio of the test mRNA to the control. All PCR product sizes were confirmed by electrophoresis on a 1.5% agarose gel and visualization using ethidium bromide.

### 2.9. Flow Cytometry

The profile of anti-AA1, PAR4, P2X7, and iNOS reactivities in the cultured BMMCs was analyzed by flow cytometry using FACSCalibur (BD Biosciences). Suspended cells were harvested from the culture plates at 4 weeks and washed with PBS by centrifugation. The cell suspensions were incubated with AA1, PAR4, P2X7, and iNOS antibodies for 30 min on ice. The cells were washed twice with ice-cold PBS and then incubated with fluorescence-conjugated secondary antibody for 60 min at 4°C in the dark. A matched isotope control was set to establish the background fluorescence. The cells were washed 3 times and then analyzed by flow cytometry. The experiment was repeated three times.

### 2.10. Statistical Analysis

All experiments were independently repeated at least three times. The values are expressed as the means ± SEMs, and the results were analyzed using an ANOVA followed by Bonferroni's post hoc test for comparisons between groups. Significance was defined by *P* values < 0.05.

## 3. Results

### 3.1. A PAR4 Agonist Inhibits the Nociceptive Response to Colorectal Distension

**Figure 1:**
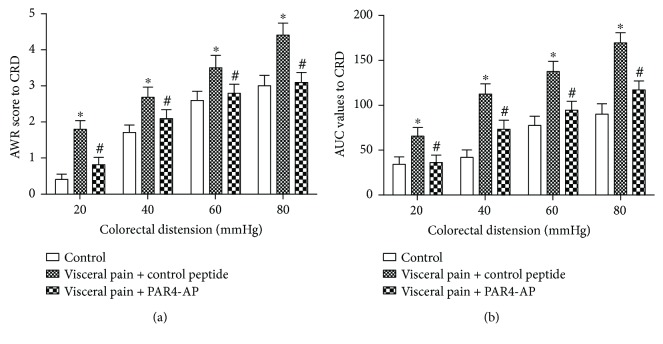
Effect of PAR4-AP on colorectal distension- (CRD-) induced visceral pain in the visceral hyperalgesia rats. (a) Abdominal withdrawal reflex (AWR) scores were used as an index of the response to CRD. (b) Area under the curve (AUC) of the electromyographic (EMG) activity in the external oblique muscle in response to CRD. All values are presented as the mean ± SEM (*n* = 6). ^∗^*P* < 0.05 versus control; ^#^*P* < 0.05 versus control peptide group.

The visceral hyperalgesia rat model was established by neonatal colorectal distention. The visceral sensitivity to CRD was determined at 8 weeks of age in the visceral hyperalgesia rats. The visceral hyperalgesia rats exhibited higher mean AWR scores and AUC values for the abdominal EMG activity at all tested distension pressures compared with the control groups (*P* < 0.05; Figures 1(a) and 1(b)). The intracolonic administration of PAR4-AP to the visceral hyperalgesia rats for 60 min elicited showed lower AWR scores and EMG activities at all tested distension pressures compared with the control peptide treatment (*P* < 0.05; Figures 1(a) and 1(b)).

### 3.2. MCs Expressing PAR4, iNOS, and P2X7 Immunoreactivity in the Colon

**Figure 2:**
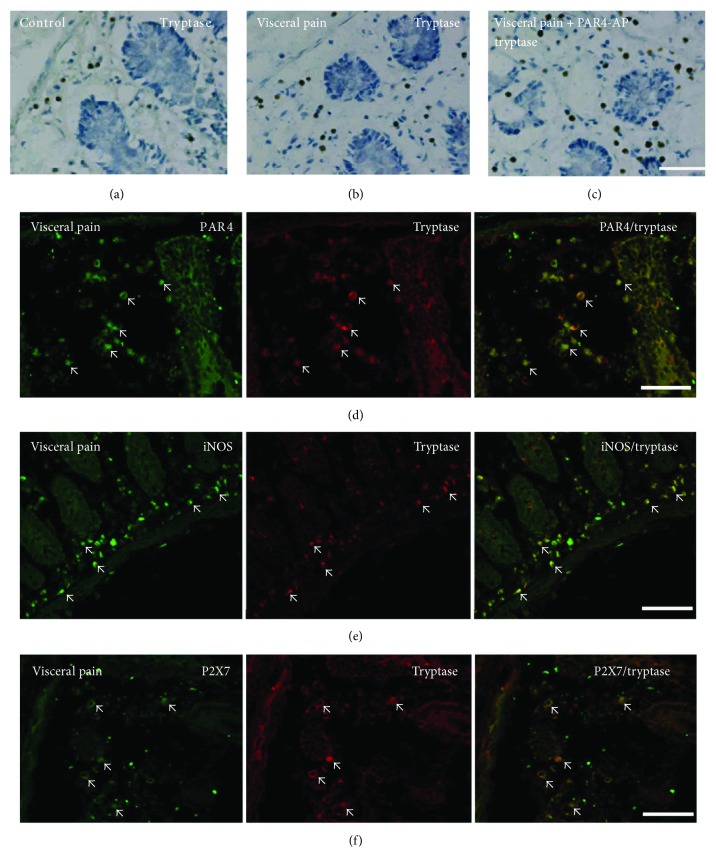
Expression of tryptase (AA1) and its colocalization with PAR4, iNOS, and P2X7 in the colonic mucosae of the visceral hyperalgesia rats. (a–c) Representative immunostainings for tryptase- (AA1-) positive MCs in the colonic sections are shown. The colonic sections were counterstained with toluidine blue. (d–f) Colonic sections from the visceral hyperalgesia rats costained with tryptase (AA1) and PAR4, iNOS, or P2X7 antibodies showing that the majority of the tryptase-positive MCs expressed PAR4, iNOS, or P2X7 (bar 100 *μ*m).

**Figure 3:**
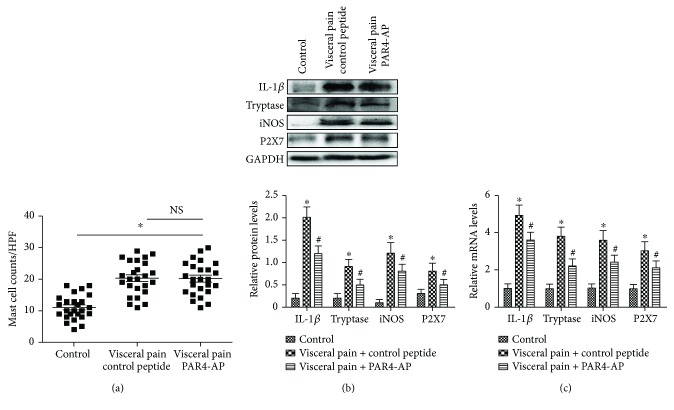
Effects of PAR4-AP on the expressions of tryptase, iNOS, P2X7, and IL-1*β* in the colons of visceral hyperalgesia rats. (a) Graph showing the numbers of tryptase- (AA1-) positive MCs in the colonic mucosae of the visceral hyperalgesia rats that were treated with PAR4-AP or control peptide (*n* = 25). HPF: high-power field. NS: no statistical significance. (b) The tryptase, iNOS, P2X7, and IL-1*β* protein levels were assessed by Western blotting. The mean optic densities of the protein were calculated by normalizing to GADPH. (c) The relative levels of tryptase, iNOS, P2X7, and IL-1*β* mRNA were measured by quantitative real-time PCR (qRT-PCR). The results were calculated by normalizing to *β*-actin in the same sample with the ΔCt method. The data are presented as the mean ± SEM (*n* = 3), ^∗^*P* < 0.05 versus controls; ^#^*P* < 0.05 versus the control peptide group.

We then analyzed the tryptase (AA1) immunopositive MCs in the colonic mucosae of the visceral hyperalgesia rats with immunohistochemistry. The number of tryptase-immunopositive MCs in the colon was significantly higher in the visceral hyperalgesia rats than in the controls (*P* < 0.05; Figures 2(a) and 2(b)). The intracolonic administration of PAR4-AP for 60 min elicited no significant difference in the number of tryptase-immunopositive MCs between the visceral hyperalgesia rats that were treated with PAR4-AP and those that were treated with the control peptide (Figures 2(b), 2(c), and 3(a)). Double labeling revealed that the tryptase-immunopositive MCs extensively expressed PAR4, iNOS, and P2X7 in the colons of the visceral hyperalgesia rats (Figures 2(d)–2(f)).

### 3.3. Effect of PAR4-AP on the Expressions of the Tryptase, iNOS, P2X7, and IL-1*β* Proteins and mRNAs in the Colon

Western blotting and qRT-PCR results revealed that the tryptase, iNOS, IL-1*β*, and P2X7 protein and mRNA levels were elevated in the colons of the visceral hyperalgesia rats compared with the controls (*P* < 0.05). Moreover, the upregulations of the tryptase, iNOS, IL-1*β*, and P2X7 protein and mRNA levels were significantly suppressed in the visceral hyperalgesia rats that were treated with PAR4-AP compared with those that were treated with the control peptide (*P* < 0.05; Figure 3).

### 3.4. Cultured Rat BMMCs Expressed Tryptase, PAR4, iNOS, and P2X7

**Figure 4:**
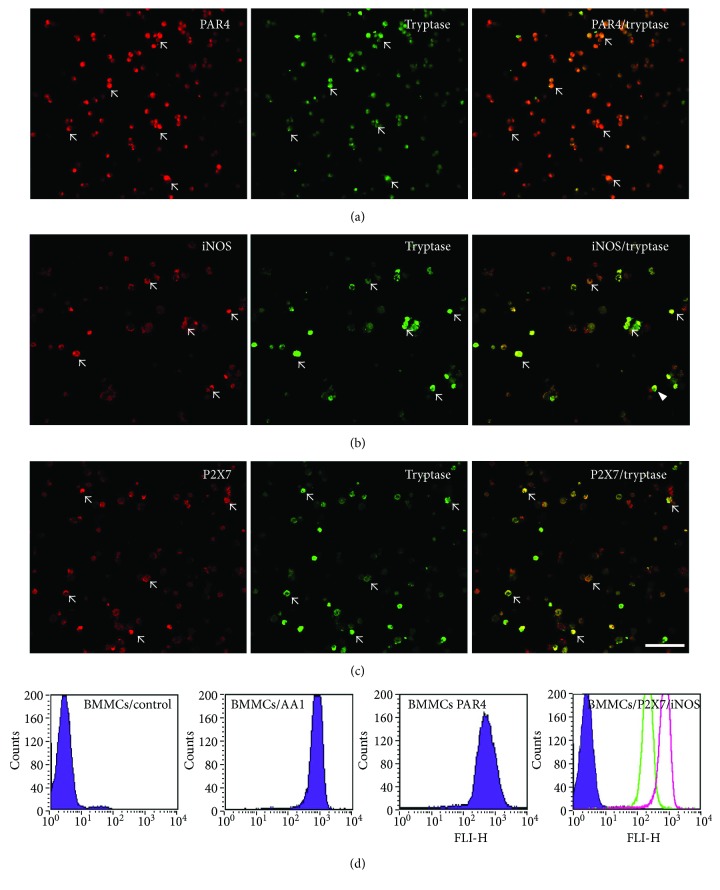
Cultured rat BMMCs expressed tryptase, PAR4, iNOS, and P2X7. (a–c) Expression of tryptase (AA1) and its colocalization with PAR4, iNOS, or P2X7 in cultured BMMCs (bar 100 *μ*m). (d) Flow cytometric analysis showed that the BMMCs expressed relatively high levels of tryptase, PAR4, iNOS, and P2X7.

Cultured BMMCs, which share some similar morphological and phenotypic properties with mucosal MCs, were prepared from the bone marrow cells of rats [19]. Immunohistochemistry for mast cell tryptase (AA1) demonstrated that 99% to 100% of the cultured BMMCs that were harvested at 4 weeks exhibited characteristics typical of MCs. Double immunofluorescence staining indicated that the vast majority of the cultured BMMCs that were harvested at 4 weeks expressed both tryptase and PAR4, iNOS, or P2X7 (Figures 4(a)–4(c)). Flow cytometric analysis indicated that cultured BMMCs that were harvested at 4 weeks expressed relatively high levels of tryptase, PAR4, P2X7 or iNOS (Figure 4(d)).

### 3.5. Effects of MAPK on Tryptase, iNOS, IL-1*β*, and P2X7 Expressions Induced by PAR4-AP in BMMCs

**Figure 5:**
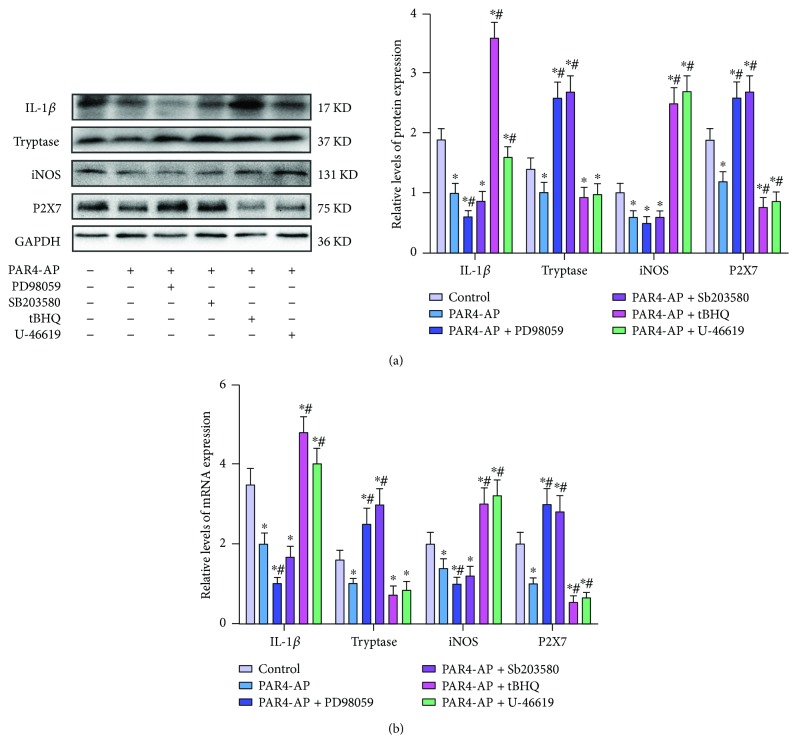
Effect of MAPK on PAR4-AP-evoked tryptase, iNOS, IL-1*β*, and P2X7 protein and mRNA expressions in BMMCs. (a) Western blotting analyses for tryptase, iNOS, IL-1*β*, and P2X7 protein expression in BMMCs following treatment with PAR4-AP and pretreatment with U-46619 (an activator of ERK1/2 and p38), tBHQ (an activator of ERK1/2), PD98059 (an inhibitor of ERK1/2), or SB203580 (an inhibitor of p38). The mean optic densities of the proteins were calculated by normalizing to GADPH. (b) Quantitative RT-PCR analyses of tryptase, iNOS, IL-1*β*, and P2X7 mRNA expression in BMMCs following treatment of the BMMCs with PAR4-AP and pretreatment with U-46619, tBHQ, PD98059, or SB203580. The results were calculated by normalizing to *β*-actin in the same sample with the ΔCt method. The changes in the relative mRNA levels are expressed as fold changes compared with the controls. All values are expressed as the means ± SEMs (*n* = 3). ^∗^*P* < 0.05 versus controls; ^#^*P* < 0.05 versus PAR4-AP-only groups.

The Western blotting and quantitative RT-PCR results revealed that the tryptase, iNOS, IL-1*β*, and P2X7 mRNA and protein levels in the BMMCs were decreased by the PAR4-AP treatment (Figure 5), which indicated that PAR4 activation decreased the tryptase, iNOS, IL-1*β*, and P2X7 expressions at both the protein and mRNA levels.

Compared with PAR4-AP alone, PD98059 and SB203580 pretreatment induced much lower iNOS and IL-1*β* protein and mRNA levels, which in turn markedly reversed the suppressive effect of PAR4 activation on the tryptase and P2X7 protein and mRNA expressions. Furthermore, tBHQ and U-46619 pretreatment induced much lower tryptase and P2X7 protein and mRNA expressions, which in turn markedly reversed the suppressive effect of PAR4 activation on the iNOS and IL-1*β* protein and mRNA levels (Figure 5). These data suggest that the role of PAR4 in suppressing the expressions of tryptase, iNOS, IL-1*β*, and P2X7 at the mRNA and protein levels was mediated by the MAPK signaling pathway.

## 4. Discussion

Our study demonstrated that the nociceptive response to CRD and the number of MCs with extensive PAR4, P2X7, and iNOS expression were increased in the colons of the visceral hyperalgesia rats. The intracolonic administration of PAR4-AP inhibited colonic hypersensitivity and reduced the expressions of tryptase, iNOS, IL-1*β*, and P2X7 in the colons of the visceral hyperalgesia rats. These effects were associated with MAPK signals that were induced by PAR4 activation. These findings provide evidence that the visceral analgesia associated with PAR4-AP may involve in downregulations of tryptase, iNOS, IL-1*β*, and P2X7 expression via MAPK signals in MCs.

Previous research suggests that PAR4 activation exerts an analgesic effect in visceral hyperalgesia through the inhibition of colonic sensory neuron excitability [9]. Furthermore, PAR4 activation has been demonstrated to reverse the PAR2 or transient receptor potential vanilloid-4 (TRPV4) activation that is evoked in colorectal hypersensitivity [8]. Moreover, the activation of PAR4 has been demonstrated to attenuate inflammatory colonic hyperalgesia in response to CRD [20]. Herein, we demonstrated that the activation of PAR4 in colonic mucosa MCs suppresses their expression of inflammatory mediators, such as tryptase, iNOS, IL-1*β*, and P2X7, which suggests that these receptors could provide additional important targets for modifying pain in colonic GI disorders, such as IBS and IBD.

Hypersensitivity is followed by the activation of the colonic MCs that are responsible for colonic barrier dysfunction [21]. Tryptase is the most abundant secretory product of MCs, an important marker of MC activation, and an important mediator of inflammation [22]. Tryptase and its cognate receptor PAR2 are involved in activating proinflammatory cytokines and colon hypersensitivity in animal models of IBD [23]. Our results demonstrated that the activation of PAR4 inhibited the expression of tryptase by colonic mucosal MCs, which might suppress the tryptase-PAR2 axis to regulate sensitization and the consequently heightened pain behavior in IBS [24].

Previous studies have demonstrated that the production of proinflammatory cytokines, such as IL-1*β*, is associated with P2X7 and NO through the iNOS pathway in MCs [25–27]. Several reports have found that increased numbers of MCs infiltrating the mucosa of the colon are correlated with the expressions of iNOS and IL-1*β* in IBS patients [13, 27], and similar findings were observed in the visceral hyperalgesia rats in the present study. The PAR4 expression in the MCs of the colonic mucosae of the visceral hyperalgesia rats that we observed was consistent with the existence of this receptor in MCs obtained from IBS patients [10, 11]. In addition to the inhibition of visceral hypersensitivity, the intracolonic administration of PAR4-AP elicited clear downregulations of iNOS, IL-1*β*, and P2X7 expression, which might have mediated the visceral hypersensitivity [12, 13]. These results illuminate a novel regulatory role of PAR4 during visceral hyperalgesia as demonstrated by the inhibited nociceptive response to colorectal distension, which was mediated by this receptor's control of inflammatory mediators (such as IL-1*β*) that were induced by NO/iNOS and P2X7 activation [25–27].

The phosphorylation of MAPK is involved in the secretion of cytokines by MCs that is induced by PAR4 activation [28]. Several studies have reported that p38 plays an important role in inflammation, and blocking p38 suppresses the transcriptional activity of NF-*κ*B and downregulates the expression of iNOS [29]. The activation of P2X7 with BZATP in murine MCs can lead to the rapid phosphorylations of ERK and p38 [30]. A previous study demonstrated that IL-1*β* can stimulate ERK1/2 and p38 phosphorylations, which upregulate proinflammatory cytokine expression [31]. The inhibition of MAPK phosphorylation by PAR4 has been found to mediate the production of inflammatory cytokines, such as IL-1*β*, in MCs, which leads to changes in nociceptive response sensitivity [32]. Our results demonstrated that PAR4 activation decreased tryptase, IL-1*β*, iNOS, and P2X7 at the protein and mRNA levels in association with the regulation of p38 and ERK1/2 phosphorylation. The suppression of these inflammatory mediators through regulation by MAPK signals after the PAR4-AP treatment of the BMMCs provided a molecular mechanism for the inhibition of visceral hypersensitivity [33, 34]. However, additional evidence is needed, and related interesting questions, for example, questions concerning interactions or crosstalk with inflammatory mediators, are worthy of further investigation.

## 5. Conclusion

This study has provided new insight into the mechanisms involved in the antinociceptive effect of PAR4 activation. A crucial role for colonic mucosal MCs in this process has been revealed. The confirmation of PAR4 expression in these cells and its role in the inhibition of tryptase, iNOS, IL-1*β*, and P2X7 expression indicates that the antinociceptive effects of PAR4-AP are linked, directly or indirectly, to MCs located in the gastrointestinal tract.
